# Study of traits and recalcitrance reduction of field-grown *COMT* down-regulated switchgrass

**DOI:** 10.1186/s13068-016-0695-7

**Published:** 2017-01-03

**Authors:** Mi Li, Yunqiao Pu, Chang Geun Yoo, Erica Gjersing, Stephen R. Decker, Crissa Doeppke, Todd Shollenberger, Timothy J. Tschaplinski, Nancy L. Engle, Robert W. Sykes, Mark F. Davis, Holly L. Baxter, Mitra Mazarei, Chunxiang Fu, Richard A. Dixon, Zeng-Yu Wang, C. Neal Stewart, Arthur J. Ragauskas

**Affiliations:** 1 BioEnergy Science Center (BESC), Oak Ridge National Laboratory (ORNL), Oak Ridge, TN USA; 2 BioSciences Division, ORNL, Oak Ridge, TN USA; 3 UT-ORNL Joint Institute for Biological Sciences, Oak Ridge, TN USA; 4 Biosciences Center, National Renewable Energy Laboratory (NREL), Golden, CO USA; 5 National Bioenergy Center, NREL, Golden, CO USA; 6 Department of Plant Sciences, University of Tennessee, Knoxville, TN USA; 7 Forage Improvement Division, The Samuel Roberts Noble Foundation, Ardmore, OK USA; 8 BioDiscovery Institute and Department of Biological Sciences, University of North Texas, Denton, TX USA; 9 Department of Chemical and Biomolecular Engineering & Department of Forestry, Wildlife and Fisheries, University of Tennessee, Knoxville, TN USA

**Keywords:** Switchgrass, Caffeic acid *O*-methyltransferase, Biomass recalcitrance, Enzymatic hydrolysis, Lignin, Cellulose accessibility

## Abstract

**Background:**

The native recalcitrance of plants hinders the biomass conversion process using current biorefinery techniques. Down-regulation of the caffeic acid *O*-methyltransferase (*COMT*) gene in the lignin biosynthesis pathway of switchgrass reduced the thermochemical and biochemical conversion recalcitrance of biomass. Due to potential environmental influences on lignin biosynthesis and deposition, studying the consequences of physicochemical changes in field-grown plants without pretreatment is essential to evaluate the performance of lignin-altered plants. We determined the chemical composition, cellulose crystallinity and the degree of its polymerization, molecular weight of hemicellulose, and cellulose accessibility of cell walls in order to better understand the fundamental features of why biomass is recalcitrant to conversion without pretreatment. The most important is to investigate whether traits and features are stable in the dynamics of field environmental effects over multiple years.

**Results:**

Field-grown *COMT* down-regulated plants maintained both reduced cell wall recalcitrance and lignin content compared with the non-transgenic controls for at least 3 seasons. The transgenic switchgrass yielded 35–84% higher total sugar release (enzymatic digestibility or saccharification) from a 72-h enzymatic hydrolysis without pretreatment and also had a 25–32% increase in enzymatic sugar release after hydrothermal pretreatment. The *COMT*-silenced switchgrass lines had consistently lower lignin content, e.g., 12 and 14% reduction for year 2 and year 3 growing season, respectively, than the control plants. By contrast, the transgenic lines had 7–8% more xylan and galactan contents than the wild-type controls. Gel permeation chromatographic results revealed that the weight-average molecular weights of hemicellulose were 7–11% lower in the transgenic than in the control lines. In addition, we found that silencing of *COMT* in switchgrass led to 20–22% increased cellulose accessibility as measured by the Simons’ stain protocol. No significant changes were observed on the arabinan and glucan contents, cellulose crystallinity, and cellulose degree of polymerization between the transgenic and control plants. With the 2-year comparative analysis, both the control and transgenic lines had significant increases in lignin and glucan contents and hemicellulose molecular weight across the growing seasons.

**Conclusions:**

The down-regulation of *COMT* in switchgrass resulting in a reduced lignin content and biomass recalcitrance is stable in a field-grown trial for at least three seasons. Among the determined affecting factors, the reduced biomass recalcitrance of the *COMT*-silenced switchgrass, grown in the field conditions for two and three seasons, was likely related to the decreased lignin content and increased biomass accessibility, whereas the cellulose crystallinity and degree of its polymerization and hemicellulose molecular weights did not contribute to the reduction of recalcitrance significantly. This finding suggests that lignin down-regulation in lignocellulosic feedstock confers improved saccharification that translates from greenhouse to field trial and that lignin content and biomass accessibility are two significant factors for developing a reduced recalcitrance feedstock by genetic modification.

**Electronic supplementary material:**

The online version of this article (doi:10.1186/s13068-016-0695-7) contains supplementary material, which is available to authorized users.

## Background

In light of the need to reduce net carbon emissions from transportation fuels and to increase energy security, cellulosic ethanol is a promising near-term alternative to fossil fuels due to its domestic abundance, renewability, and favorable net carbon emissions [1, 2]. Switchgrass (*Panicum virgatum* L.) is a dedicated lignocellulosic biofuel feedstock in the USA, due to its high productivity, flexible adaptability to existing agricultural operations, and low agronomic input requirements [3, 4]. Like other lignocellulosic materials, however, switchgrass cell wall is a complex composite consisting primarily of three biopolymers: cellulose, hemicelluloses, and lignin. The evolved heterogeneity and complexity of plant cell wall structure and components impart natural resistance to enzymatic and microbial deconstruction on lignocellulosic bioresources, which is termed as recalcitrance [5, 6]. Low biomass recalcitrance favors the economics of the biorefinery.

The recalcitrance of lignocellulosic biomass has been attributed to several factors, such as accessible surface area, lignin content and structure, cellulose crystallinity and degree of polymerization (DP), and hemicellulose content, as well as the presence of acetyl groups [7]. In particular, lignin, a phenolic polymer primarily composed of *p*-hydroxyphenyl (H), guaiacyl (G), and syringyl (S) units, has been regarded as one of the major contributors to recalcitrance by restricting accessibility of enzymes to cell wall polysaccharides and non-productively binding to cellulases [8, 9]. To overcome the recalcitrance derived from cross-linked lignin and polysaccharide networks, a pretreatment is generally required to break down the lignin–carbohydrate matrix and increase the enzymatic digestibility of cell wall polysaccharides [10]. The drawbacks of the pretreatment process are typically their capital expense, energy costs, and the generation of inhibitors to the subsequent microbial fermentation process in the current bioethanol production platform [10, 11].

An alternative promising approach to reduce biomass recalcitrance is to develop genetically engineered feedstock that are more susceptible to pretreatment and eventually to chemical, enzymatic, and microbial digestion [6, 12, 13]. Reduction of recalcitrance has been achieved by altering lignin content and composition through manipulating genes encoding enzymes involved in the lignification process [13]. Caffeic acid/5-hydroxyconiferyl aldehyde *O*-methyltransferase (*COMT*) functions in the synthesis of S monolignol units by catalyzing the *O*-methylation of the 5-hydroxyl groups of the monolignol precursors 5-hydroxyconiferyl aldehyde and 5-hydroxyconiferyl alcohol [14, 15]. The suppression of *COMT* activity in switchgrass by RNAi-mediated gene silencing results in a decrease in total lignin content and S units in biomass, a normal growth phenotype, and improved forage quality [12]. Compared with non-transgenic switchgrass controls, the greenhouse-grown transgenic switchgrass exhibited increased saccharification yield by 17–22% with pretreatment and 29–38% without pretreatment, and increased ethanol production yield by 14–28% in simultaneous saccharification and fermentation (SSF) or by 18% in consolidated bioprocessing (CBP). In contrast, fermentation inhibition of hot water pretreated *COMT*-deficient switchgrass without water washing was observed compared with wild-type controls, due to the potential accumulation of monolignol-like metabolites [16]. Transgenic switchgrass grown under greenhouse conditions had decreased S/G and *p*-coumarate/ferulate ratios [17]. However, the cellulose crystallinity index (CrI) and DP of transgenic switchgrass were identical to those of controls [12]. A recent study [18] of a 2-year field trial showed that the reduced lignin content of the *COMT* down-regulated switchgrass was maintained when plants were grown in the field, associated with increased saccharification yield and ethanol yield by up to 34 and 28%, respectively. However, the potential influences of factors such as biomass accessibility or the presence of hemicellulose on the reduced recalcitrance of *COMT* down-regulated switchgrass have not been examined.

For a better understanding of the structural properties of *COMT* down-regulated plants grown under field conditions, several factors, including lignin content, cell wall accessibility, cellulose crystallinity, cellulose DP, and molecular weight of hemicellulose, were determined and correlated empirically with enzymatic digestion efficacy. The *COMT* transgenic switchgrass and their controls (non-transgenic switchgrass) from the previous greenhouse study [12] were field-grown and harvested in year 1 to year 3 [18, 19]. The field analysis had shown that the *COMT2* transgenic line grown in the field exhibited a significant feedstock improvement by yielding over 50% more liters of ethanol per hectare [18]. The authors also suggested that the plants had reached a more steady-state growth pattern in the second year after the first year of establishment. Therefore, the *COMT2* transgenic line and its control harvested in 2012 (year 2) and 2013 (year 3) were used in the present study. The main objectives of this study were to (1) assess the sugar release efficiency and characterize the structural differences of field-grown transgenic switchgrass and control, and (2) determine the stability of a few cell wall recalcitrance-related traits and their importance to the enhanced saccharification efficacy in *COMT* down-regulated switchgrass without pretreatment.

## Results

### Chemical composition of switchgrass

**Fig. 1:**
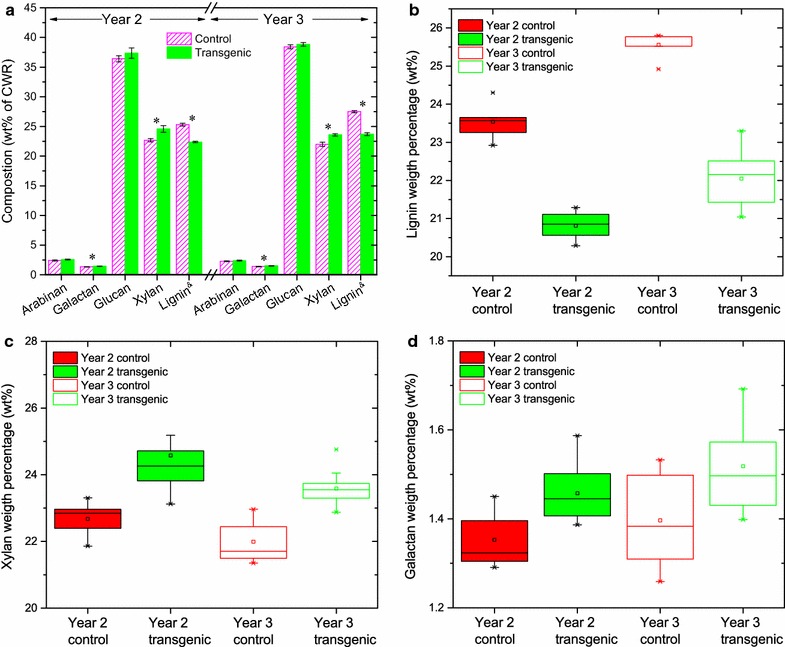
Chemical composition of field-grown switchgrass in years 2 and 3. The values (wt% of cell wall residue) reported are the average of 5 biological replicates from each control group and 10 biological replicates from each transgenic group (**a**). *Error bars* represent standard errors. Mannan was not detectable. An *asterisk* (*) indicates a significant difference between the transgenic and control groups as determined by a Student’s *t* test (*P* < 0.05). The distributions of lignin (**b**), xylan (**c**), and galactan (**d**) contents in switchgrass were compared. ^&^ The lignin content data of year 2 and year 3 plants are from previous publications [18, 19]

Both the transgenic and control plants were primarily composed of glucan, xylan, and lignin, as well as minor components such as arabinan and galactan (Fig. 1a). Mannan was not detectable in these plants. The distribution of the three significantly changed traits: lignin, xylan, and galactan contents after the 2-year growing period, were compared and are shown in Fig. 1b–d separately. The *COMT* down-regulated year 2 and year 3 field-grown switchgrass had 12% (*P* < 0.0001) and 14% (*P* < 0.0001) less lignin, respectively, compared with the controls (Fig. 1b). This reduction in lignin content is consistent with previous results observed for both the greenhouse-grown (11–13%) [12, 17] and the field-grown (11–14%) [18, 19] *COMT* down-regulated switchgrass. In addition, the down-regulation of *COMT* resulted in slightly higher polysaccharide content including a 7–8% increase in xylan (*P* < 0.01) and an 8% increase in galactan (*P* = 0.03) compared with the controls (Fig. 1c, d). The xylan and galactan contents in transgenic switchgrass of both years 2 and 3 were significantly higher than those in the corresponding controls, whereas this difference was not observed in the field-grown year 1 switchgrass [18]. This variation agreed with the greenhouse-grown switchgrass with a 5–6% higher xylan content in T_1_ generation *COMT* down-regulated lines relative to their controls [12, 17].

After the first-year establishment in the field, chemical compositions of the field-grown switchgrass, lignin and glucan contents, varied across the plants’ 2-year rotation cycles. Along with the increased biomass yields in Table 1 [19], the lignin contents of the control and transgenic lines increased, by 9 and 6% (*P* < 0.0001), respectively, in year 3 compared with those in year 2 (Fig. 1b). In addition, we found that year 3 plants also showed a 4–5% increase (*P* < 0.05) in glucan content with respect to year 2. The contents of the other measured polysaccharides, arabinan, galactan, and xylan, did not change significantly over the 2-year study period (Additional file 1: Table S1, S6).

**Table 1:** Biomass yields, dyes adsorption on switchgrass, DP of cellulose, and molecular weight of hemicellulose

	Biomass yield	Cellulose accessibility (mg/g)			Cellulose			Hemicellulose (×10^3^ g/mol)	
	*Y*_DW_ (kg/m^2^)	*A*_O_	*A*_B_	O/B	DP_n_	DP_w_	PDI	*M*_w_	*M*_n_
Reference		24.6^a^	9.3^a^	2.65^a^	39 ± 4^a^	283 ± 12^a^	7.3^a^	18.7 ± 0.7^b^	10.8 ± 0.6^b^
Year 2 control	2.2 ± 0.2	5.4	5.4	1.02	431 ± 25	3729 ± 183	8.7	31.6 ± 3.0	21.6 ± 3.7
Year 2 transgenic	2.6 ± 0.1*	6.6*	5.0	1.35	438 ± 50	3855 ± 299	8.9	29.3 ± 1.9*	19.7 ± 2.2
Year 3 control	2.5 ± 0.1	5.3	5.5	0.98	421 ± 52	3848 ± 131	9.2	36.0 ± 1.2	23.7 ± 0.9
Year 3 transgenic	2.9 ± 0.1	6.4*	6.2	1.09	402 ± 44	3875 ± 187	9.7	32.1 ± 3.0*	21.1 ± 1.6*

*Y*_DW_ is the dry weight biomass yield adapted from literature [19]. The maximum adsorption capacity of orange (*A*_O_) and blue (*A*_B_), adsorption ratio of orange to blue (O/B), degree of polymerization (DP) and polydispersity index (PDI) of cellulose, and the average molecular weights of hemicellulose of *COMT* down-regulated switchgrass and controls. The value reported was the average of 5 biological replicates from each control group and 10 biological replicates from each transgenic group. DP_n_ and DP_w_: number-average and weight-average degree of polymerization; *M*_n_ and *M*_w_: number-average and weight-average molecular weights; a: Avicel PH101; b: beech wood xylan. An asterisk (*) for the bold numbers indicates a significant difference between the transgenic and control groups as determined by a Student’s *t* test (*P* < 0.05). ± is followed by standard deviation

### Enzymatic digestibility of hydrothermally pretreated and unpretreated switchgrass

**Fig. 2:**
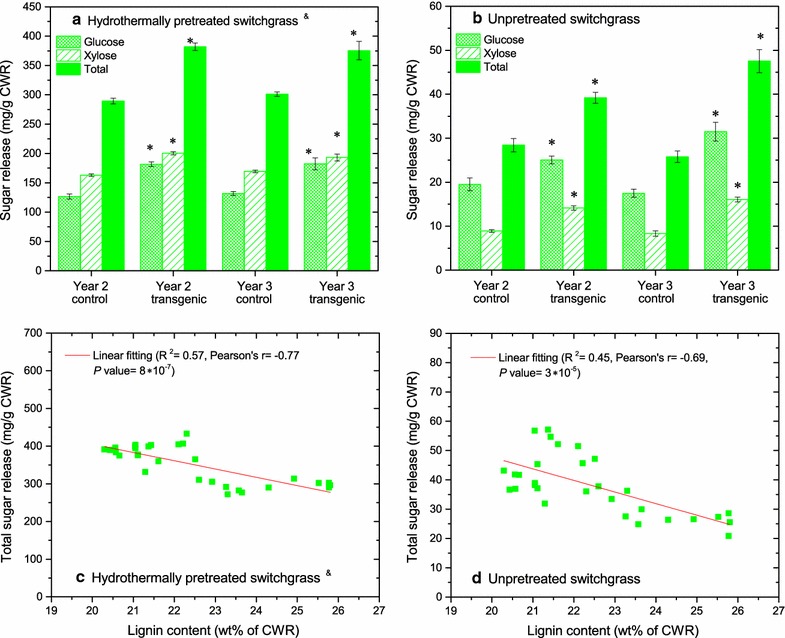
Sugar release and its relationship to lignin content. Total sugar release (mg per g cell wall residues) from hydrothermally pretreated (**a**) and unpretreated (**b**) switchgrass in years 2 and 3 after 72-h enzymatic hydrolysis (the value reported is the average of 5 biological replicates from each control group and 10 biological replicates from each transgenic group. *Error bars* represent standard errors.) and the relationship of total sugar (glucose and xylose) release for pretreated (**c**) and unpretreated (**d**) switchgrass to lignin content (wt% of cell wall residues). An *asterisk* (*) indicates a significant difference between the transgenic and control groups as determined by a Student’s *t* test (*P* < 0.05). ^&^ The sugar release and lignin content data of year 2 and year 3 plants after pretreatment are from a previous publication [18, 19]

The hydrothermally pretreated (also called autohydrolysis or hot water pretreatment) transgenic samples grown in the field exhibited higher saccharification yield than controls (Fig. 2a) [18, 19]. We found that the unpretreated transgenic line in both years 2 and 3 exhibited significant increases in saccharification yield with 38 and 84% higher total sugars released (*P* < 0.0001), respectively, compared with the controls (Fig. 2b). The sugar release improvement in year 2 plants (38%) was comparable to the greenhouse-grown switchgrass (29–38%) [12], whereas that in year 3 switchgrass was improved by 84%. On the basis of sugars present in biomass, field-grown switchgrass had a total sugar release of 4–7%, whereas the greenhouse-grown switchgrass (green tissues) demonstrated higher sugar releases of about 20–30% [12].

The enzymatic digestibility of lignocellulosic biomass has been thought to be strongly correlated with lignin content [8, 20]. By correlating the total sugar release with the lignin content in switchgrass, we found that sugar release of hydrothermally pretreated plants decreased with the lignin content with *R* ^2^ = 0.57 (Pearson’s *r* = −0.77, *P* value = 8 × 10^−7^) (Fig. 2c). Similarly, an inverse linear relationship with *R* ^2^ = 0.47 (Pearson’s *r* = −0.69, *P* value = 3 × 10^−5^) of sugar release of unpretreated switchgrass to lignin content was also observed (Fig. 2d).

### Cellulose accessibility estimation by Simons’ stain

**Fig. 3:**
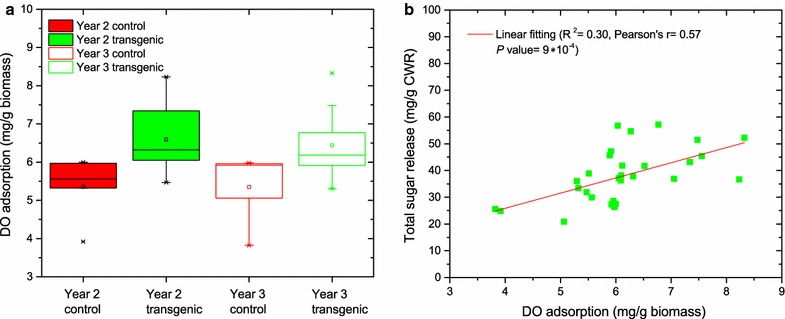
Distribution of DO adsorption and its relationship to sugar release. The distribution of orange dye adsorption (*A*_O_) on unpretreated biomass measured by Simons’ stain (**a**) and its relationship to the total sugar release (glucose and xylose) for unpretreated switchgrass (**b**)

Simons’ stain measures the competitive adsorption between the direct orange (DO) dye 15 and the direct blue (DB) dye 1 on a cellulosic substrate and has been used as a semi-quantitative and relatively easy method to assess the “overall” accessible surface area [21, 22]. To assess the accessibility of unpretreated switchgrass, the adsorption capacities of DO and DB on both transgenic and control plants were measured and compared (Table 1). Avicel PH-101, with 25 and 9 mg/g maximum adsorption of DO and DB, respectively, was a positive control and gave values consistent with previous results [23]. The transgenic switchgrass in both years 2 and 3 had a significantly higher (20–22%, *P* < 0.05) adsorption capacity of orange A_O_ than its control. However, there was no significant difference observed with respect to A_O_ across the investigated growing seasons (Fig. 3a). The measured A_O_ adsorption was significantly smaller on unpretreated biomass than the pretreated biomass reported in [22] likely due to the cell wall matrix in pretreated biomass being disrupted by pretreatment leading to increased cellulose exposure. Significantly lower DO adsorption was also reported on unpretreated poplar [24, 25]. In addition, the switchgrass adsorbed more orange than blue and the O/B ratio was higher in the transgenic (1.35 and 1.09) relative to the control (1.02 and 0.98) group. A positive relationship (*R* ^2^ = 0.30, Pearson’s *r* = 0.57, *P* < 0.001) was observed between the sugar release and DO adsorption on unpretreated biomass (Fig. 3b).

### Molecular weight of cellulose and hemicellulose and crystallinity of cellulose

**Fig. 4:**
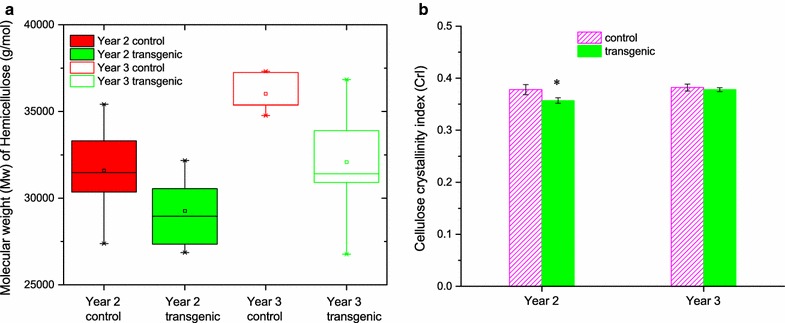
Hemicellulose molecular weight distribution and cellulose crystallinity index (CrI). Distribution of weight-average molecular weight (*M*_w_) of hemicellulose (**a**) and the average CrI of cellulose (**b**). CrI values reported are the average of 5 biological replicates from each control group and 10 biological replicates from each transgenic group. *Error bars* represent standard errors. An *asterisk* (*) indicates a significant difference between the transgenic and control groups as determined by a Student’s *t* test (*P* < 0.05)

The number-average degree of polymerization (DP_n_) and the weight-average degree of polymerization (DP_w_) of cellulose from switchgrass were in the range of 400–450 and 3700–3900, respectively (Table 1). Comparable with the values (500–700 and 3300–4200 for DP_n_ and DP_w_, respectively) determined for greenhouse-grown switchgrass [12], the cellulose DP of field-grown transgenic plants did not show significant variation from that of their controls nor changes across growing seasons based on a Student’s *t* test (Additional file 1: Table S5, S6). The extracted hemicellulose from the switchgrass had a *M*_n_ of 2.0–2.4 × 10^4^ g/mol and a *M*_w_ of 2.9–3.6 × 10^4^ g/mol, and the transgenic plants had 7% (*P* < 0.05) and 11% (*P* < 0.01) reduction in M_w_ compared with their controls in years 2 and 3, respectively (Table 1; Fig. 4a). The sugar release showed an inverse dependence on the molecular weights of hemicellulose (Additional file 1: Figure S3). The average crystallinity index (CrI) of cellulose isolated from switchgrass from years 2 and 3 were similar, in the range of 36–38% (Fig. 4b); this similarity between transgenic and control switchgrass was also observed for the greenhouse-grown plants [12].

## Discussion

### *COMT* down-regulation sustained reduced lignin and indirect impact on polysaccharide contents

The chemical composition of biomass is an important factor for biomass utilization as well as its digestibility [7]. As greenhouse-grown *COMT* down-regulated switchgrass has exhibited a significant reduction of lignin content and biomass recalcitrance, the variation of their chemical composition and performance occurs when plants are subjected to field trials with extended environmental stresses [18, 26]. The lignification (and biomass recalcitrance) may be influenced by abiotic (e.g., temperature changes, light and oxidative stress, water deficiency, etc.) and biotic (e.g., pathogen infection) stresses [27]. Our results showed that the field-grown switchgrass had a similar chemical composition as the same cultivated line grown in a greenhouse. Compared with the control plants, the reduced lignin content of *COMT* down-regulated switchgrass was maintained in the field for the first three seasons. Interestingly, lignin content in both the transgenic and control switchgrass lines showed a significant increase across the three harvest years when grown in the field (e.g., 18.1 and 20.4% in year 1, 20.8 and 23.5% in year 2, and 22.1 and 25.6% in year 3, respectively) [18, 19]. The lignin content increase in field-grown switchgrass from year 1 to year 2 is expected given the longer time available for lignification in the second growing season. The further increase between years 2 and 3 is also expected given the relationship between biomass (plant size revealed by biomass yields in Table 1) and lignification as the plants grow older. In addition, both the year 2 and year 3 field-grown transgenic lines had different polysaccharide contents from the control plants. This difference was mainly caused by 7–8% higher xylan and 7–8% higher galactan contents, whereas the largest polysaccharide proportion in biomass, glucan content, was comparable between the transgenic and control plants. The xylan content increase, 14% in control and 22% in *COMT* lines, was also observed in year 2 in comparison to year 1 [18]. Therefore, *COMT* down-regulation in switchgrass also had an indirect increase of hemicellulose in the plant cell wall.

### Improved sugar release preserved in the field-grown transgenic switchgrass

The hydrothermal pretreatment is one of the promising technologies to reduce biomass recalcitrance by breaking down plant cell wall intricacy and making polysaccharides more accessible and amenable to enzymes [28]. The amount of sugars released from the plant cell wall is then used to estimate the biomass recalcitrance. The improvement of total sugar release (6% in year 1, 32% in year 2, and 25% in year 3) from the transgenic switchgrass relative to the control plants revealed that the *COMT* down-regulated switchgrass grown in the field maintained the reduced biomass recalcitrance (Fig. 2a) [18, 19]. We also observed the enhanced sugar release from unpretreated biomass and the higher saccharification yield indicating the reduced recalcitrance of *COMT* down-regulated switchgrass (Fig. 2b). Transplanted from the greenhouse, the switchgrass growing in the field responds and adapts to a range of stresses including pathogens, temperature and light fluctuations, and water deficits. The enzymatic hydrolysis is more efficient for younger tissue grown in the greenhouse [12] than for senesced tissue harvested from the field experiments, which is in agreement with a previous finding [29]. Taking the improved enzymatic digestibility of transgenic switchgrass with hydrothermal pretreatment and without pretreatment together, the reduced recalcitrance of the *COMT* down-regulated switchgrass grown under the field conditions has been retained for at least 3 years.

### Traits changes and their importance to cell wall recalcitrance

Structural heterogeneity and complexity of cell wall components, such as the matrix and structures of lignin, cellulose, and hemicellulose, were thought to be factors contributing biomass recalcitrance [6, 7]. Lignin contributes to biomass recalcitrance primarily by limiting enzyme access to polysaccharides through physical shielding [30] and/or unproductive binding to enzymes [31]. To assess the association of the lignin with reduced recalcitrance in the *COMT* down-regulated switchgrass, lignin content was correlated with the sugar release of hydrothermally pretreated switchgrass. A dependence of sugar release on lignin content was observed in our study (Fig. 2c). Studer et al. [20] observed a similar trend in that the lignin content in natural *Populus trichocarpa* variants negatively correlated with sugar release. Since a potential variation of lignin could occur during pretreatment (i.e., lignin fragmentation and removal) [28], the correlation of sugar release and lignin content in unpretreated biomass was also investigated (Fig. 2d). The inverse linear correlation of enzymatic digestibility of both unpretreated and hydrothermally pretreated switchgrass and lignin content suggests that the reduced biomass recalcitrance of *COMT* down-regulated switchgrass was likely governed by the lignin content.

Simons’ stain has been employed to assess the substrate accessibility to enzymes due to the good linear correlation between the Simons’ stain results and the enzymatic digestibility of biomass [32, 33]. The 20–22% higher adsorption capacity of orange (*A*_O_) of unpretreated transgenic switchgrass than controls in years 2 and 3 indicates that transgenic switchgrass had relatively larger cellulose accessible regions that could more readily be attached to by cellulase. In addition, the switchgrass adsorbed more orange than blue, and the O/B ratio was higher for the transgenic relative to the control plants (Table 1). Due to the size difference of DB (<5 nm) and DO (5–36 nm) [21], O/B could be used to indicate the relative proportions of pore sizes of the substrate. Considering the higher affinity of DO than DB on lignocellulose, higher O/B values indicate a relatively greater proportion of larger porous regions within the transgenic switchgrass that will be mostly accommodated by DO, while the small pores could only be accessed by DB. Hence, higher *A*_O_ and O/B values in the transgenic plants indicate that field-grown *COMT*-deficient switchgrass possessed more cellulose accessibility than control plants. Owing to the higher affinity of DO for substrate and the similar size of orange molecular to cellulases (e.g., *Trichoderma reesei* 4 × 5 × 6 nm^3^), the orange adsorption capacity by itself can be used to assess the enzyme accessibility and, therefore, susceptibility of biomass [22, 34]. The positive correlation of DO adsorption capacity and sugar release of biomass (Fig. 3b) implied that reduced biomass recalcitrance could be associated with larger biomass accessibility. The biomass with higher adsorption of DO which is attributed to larger pore structure facilitates the accommodation and penetration of enzymes during polysaccharide hydrolysis. However, the correlation between saccharification efficiency and cellulose accessibility of unpretreated switchgrass was much lower than pretreated biomass [22]. This result of relationship is consistent with the correlation of unpretreated poplar with glucose release (*R* ^2^ = 0.33) [24]. Although statistically different, the increase of cellulose accessibility in the unpretreated biomass is still in a small range and could be easily sheltered by the complexity of cell wall structure.

Other factors associated with biomass recalcitrance include DP and crystallinity of cellulose and the properties of hemicellulose [7]. The DP of cellulose in either a relatively pure cellulosic substrate or lignocellulosic biomass has been thought to be one of the important factors affecting the efficiency of enzymatic hydrolysis [35]. Similar to the greenhouse-grown switchgrass [12], the cellulose DP of transgenic plants did not show significant variation from that of their controls. The crystallinity index (CrI) has been reported to be linearly proportional to the saccharification efficiency of cellulosic substrates, e.g., the CrI of Avicel was directly related to hydrolysis rate by cellulases [36]. The similarity of cellulose CrIs between transgenic and control switchgrass was also observed for the greenhouse-grown switchgrass, although the absolute values of CrIs are relatively lower, compared with those from greenhouse-grown plants (52-55%) [12]. However, no correlation was found between enzymatic digestibility and cellulose DP (Additional file 1: Figure S2) or CrIs (Additional file 1: Figure S4) in our study. Therefore, the small variation of cellulose CrI and DP between the transgenic and control lines suggests that cellulose CrI and DP are not contributing to the reduced recalcitrance of the field-grown COMT switchgrass.

Hemicelluloses are considered to be another factor contributing to biomass recalcitrance by limiting enzyme accessibility as a physical barrier [7]. It is interesting to find that the *COMT* down-regulation caused reduced hemicellulose molecular weights in the field-grown switchgrass. The molecular weight of hemicellulose was widely distributed depending on extraction conditions. Ayoub et al. [37] reported that the extraction of switchgrass hemicellulose with 18% NaOH at 50 °C for 3 h resulted in a much lower *M*_n_ of 460 g/mol and *M*_w_ of 3500 g/mol due to alkaline degradation. Thus, our hemicellulose was extracted at lower temperature (~22 °C) and shorter time (4 h) to avoid significant degradation. Previous study has found that the high lignin content in biomass limits the extraction of hemicellulose, likely because of the presence of lignin–carbohydrate complex linkages [38]. Although the reduction of hemicellulose molecular weight in the transgenic lines is interesting, the potential reason cannot be confirmed without further investigation. Together with the changes in hemicellulose contents, our results imply that the *COMT* down-regulation had a greater influence on hemicellulose than cellulose in switchgrass cell walls. In addition, hemicellulose in transgenic switchgrass with lower *M*_n_ and *M*_w_ tends to have shorter chains and more reducing ends, which are beneficial to exo-xylanases. However, no correlation between the hemicellulose molecular weights and sugar release (Additional file 1: Figure S3) suggests that the structural change of hemicellulose is not likely to be the major contributor to the reduced recalcitrance of the *COMT* down-regulated switchgrass.

## Conclusions

The current study reports the differences and stability of several plant traits of plants without pretreatment: *COMT*-suppressed switchgrass and wild-type control with two growing seasons (years 2 and 3) in field trials. Overall, the reduced lignin content and recalcitrance of the transgenic plants are durable in the field for at least 3 years. Silencing of *COMT* in switchgrass led to increased cellulose accessibility, measured by Simons’ stain. The manipulation of lignin biosynthesis in switchgrass also had an indirect influence on hemicellulose in plants demonstrated by increased xylan and galactan contents and decreased hemicellulose molecular weights. Over the latter 2-year field observation, lignin content and hemicellulose molecular weights increased significantly. The reduced lignin content and increased cell wall accessibility are the two important factors of those measured traits, which are likely to be associated with the reduced recalcitrance of the *COMT*-silenced field-grown switchgrass. The comparative observations and analysis results in this study provide a mechanistic understanding of cell wall recalcitrance in lignin-reduced mutants.

## Methods

### Chemicals

The enzyme cocktail Cellic CTec2 was obtained from Novozyme (Franklinton, NC). Direct Blue (DB) 1 (Pontamine Fast Sky Blue 6BX) and Direct Orange (DO) 15 (Pontamine Fast Orange 6RN) dyes were obtained from Pylam Products Co. Inc. (Garden City, NY). Peracetic acid solution (32 wt% in dilute acetic acid), phenyl isocyanate (assay grade), and dichloromethane (HPLC grade) were purchased from Sigma-Aldrich (St. Louis, MO). Anhydrous pyridine (EMD, Millipore) was purchased from VWR. All the reagents and chemicals, unless otherwise noted, were used as received.

### Generation and growth of transgenic and control switchgrass

In a previous study, a lowland-type switchgrass (cv. Alamo) was genetically modified by down-regulating *COMT* gene expression by RNAi-mediated gene silencing [12]. A scheme of the proposed lignin biosynthesis pathway involving *COMT* is shown (Additional file 1: Figure S1). Briefly, a T_0_ event was crossed with a non-transformed ‘Alamo’ accession to produce the T_1_ generation. The 10 plants of the T_1_-generation *COMT2* line are biological replicates, each grown from separate seeds resulting from a cross between the T_0_-generation COMT2 event and an Alamo wild-type plant. A population of the T_1_ transgenic switchgrass and its T_1_ null-segregant controls were grown in Knoxville, Tennessee, USA. The 3-year experiment from this field has been described elsewhere [18, 19]. The field trial was established in 2011, and the aboveground senesced biomass was harvested after a killing frost in December of each year from 2011 to 2013. In the current study, ten transgenic replicates for *COMT2* and five corresponding null-segregant (non-transgenic control) replicates in 2012 (year 2) and 2013 (year 3) were selected for analysis. All senesced tissue samples were ground with a Wiley mill to 0.8–1.0 mm after oven-drying (43 °C, 96 h) before further analysis.

### Chemical compositional analysis

The compositional analysis of switchgrass was performed as described previously [18]. Briefly, the extractive-free samples, designated as cell wall residues (CWR) in this study, were subjected to a two-step hydrolysis according to the protocol developed by NREL (http://www.nrel.gov/docs/gen/fy08/42623.pdf) using three replicates. The monomeric sugar units were quantified via a Flexar high-pressure liquid chromatography (HPLC) column (Perkin Elmer, Shelton, CT) equipped with a refractive index (RI) detector, an Aminex HPX-87P carbohydrate column, and a de-ashing guard column from Bio-Rad (Hercules, CA). The lignin contents of all biomass samples were analyzed by pyrolysis molecular beam mass spectrometry (Py-MBMS), as described elsewhere [18, 39].

### Enzymatic hydrolysis and sugar release

Enzymatic hydrolysis of unpretreated and the resultant pretreated switchgrass was carried out in 96-well reactor plates using a high-throughput pretreatment and hydrolysis technique (HTPH) [40]. In brief, 5.00 ± 0.30 mg extractive-free switchgrass was loaded into custom 96-well Hastelloy reactor plates. After the addition of 250 μL of water to each well, the reactor plates were sealed and pretreated for 17.5 min at 180 °C in a modified two-gallon Parr reactor (model 4554, Parr Instrument Company). After cooling, 40 mL of CTec2 cellulase diluted in 1.0 M citrate buffer pH 5.0 was added to a final loading of 70 mg protein/g glucan. After resealing the reactor plates, enzymatic hydrolysis was performed at 50 °C for 70 h. The released glucose and xylose contents in the supernatant were quantified by sugar-specific oxidation–reduction assays [40]. If pretreatment was omitted, the samples were soaked for 4 h in water and then directly subjected to enzymatic hydrolysis with CTec2 as described. For each independent biomass sample, three analytical replicates were performed. The enzymatic digestibility (or saccharification yield) was reported in terms of the sugar release amount as mg sugar per g CWR.

### Isolation of cellulose and hemicellulose

Cellulose and hemicellulose were isolated from switchgrass biomass according to published procedures [41, 42]. The extractive-free samples were delignified by peracetic acid with 5.00 g loading per g biomass. The solution consistency was adjusted to 5% with deionized (DI) water and the holopulping was conducted at room temperature for 24 h with magnetic stirring. The solid residue, designated as holocellulose, was washed with excessive DI water (18.0 MΩ) and air dried at room temperature for 24 h. A sub-portion of the air-dried holocellulose (100 mg) was consecutively extracted at 25 °C with 17.5% (wt/vol) NaOH solution (5.00 mL) for 2 h, followed by 8.75% NaOH solution (10.00 mL) for an additional 2 h. The alkaline slurry was then filtered and rinsed with 5 mL of 1% acetic acid leading to a liquid fraction and a solid residue. The solid residue, namely α-cellulose, was washed with an excess of DI water and air dried for the analysis of cellulose DP. The liquid fraction, rich in hemicellulose, was adjusted to pH 6–7 with anhydrous acetic acid. Hemicellulose was then precipitated by adding three volumes of 100% ethanol to the liquid fraction. Hemicellulose was then obtained by centrifugation at 8000 rpm (267 π rad/s) for 5 min and freeze dried for 24 h.

### Gel permeation chromatographic (GPC) analysis

The weight-average molecular weight (*M*_*w*_) and number-average molecular weight (*M*_*n*_) of cellulose were measured by GPC after tricarbanilation, as described previously [41]. Briefly, α-cellulose was derivatized with phenyl isocyanate in an anhydrous pyridine system prior to GPC analysis. Size-exclusion separation was performed on an Agilent 1200 HPLC system (Agilent Technologies, Inc, Santa Clara, CA) equipped with Waters Styragel columns (HR1, HR4, and HR5; Waters Corporation, Milford, MA). Number-average degree of polymerization (DP_n_) and weight-average degree of polymerization (DP_w_) of cellulose were obtained by dividing *M*_n_ and *M*_w_, respectively, by 519 g/mol, the molecular weight of the tricarbanilated cellulose repeating unit. The molecular weights of hemicellulose were measured by an Agilent 1200 series HPLC system equipped with three columns of Ultrahydrogel 120, 250, and 500 (Waters Inc.) linked in series according to [43]. The freeze-dried hemicellulose samples were dissolved in 0.2 M sodium hydroxide/0.1 M sodium acetate (pH 13.0) mobile phase (~1.0 mg/mL) directly and filtered through a 0.45-µm filter before GPC analysis.

### Solid-state NMR analysis

Solid-state NMR analysis for cellulose crystallinity was performed as described previously with minor modification [42]. The isolated cellulose samples were stored in a sealed container to prevent moisture loss. The NMR samples were prepared by packing the moisturized cellulose into 4-mm cylindrical Zirconia MAS rotors. Cross-polarization magic angle spinning (CP/MAS) NMR analysis of cellulose was carried out on a Bruker Advance-400 spectrometer operating at a frequency of 100.59 MHz for ^13^C in a Bruker double-resonance MAS probe head at the spinning speed of 10 kHz. CP/MAS experiments utilized a 5 µs (90°) proton pulse, 1.5 ms contact pulse, 4 s recycle delay, and 4000 scans. The cellulose crystallinity index (CrI) was determined from the areas of the crystalline and amorphous C_4_ signals using the following formula:

$$ {\text{CrI}} = \frac{{A_{{ 8 6 -  9 2 {\text{ ppm}}}}  }}{{A_{{ 8 6 -  9 2 {\text{ ppm}}}} \, + \,A_{{79 - 86\,{\text{ppm}}}} }}. $$

### Simons’ stain

The accessibility of unpretreated switchgrass was estimated by a down-scaled Simons’ stain procedure with DB 1 and DO 15 dyes, as described previously [22, 34]. In detail, extractive-free switchgrass (10.00 mg oven-dry basis) was weighed into seven micro-centrifuge tubes (2 mL) individually. Phosphate-buffered saline solution (pH 6.0, 0.30 M PO_4_^3-^, 1.40 mM NaCl) (0.10 mL) was added to each tube. A set of dye solutions containing 0.20, 0.40, 0.60, 0.80, 1.20, 1.60, and 2.00 mg/mL of both DO and DB was made and 0.50 mL of the solution was added to each tube. DI water (0.40 mL) was added to each tube to make up the final volume to 1.00 mL to give concentrations of DO and DB of 0.10, 0.20, 0.30, 0.40, 0.60, 0.80, and 1.00 mg/mL. The centrifuge tubes were incubated at 70 °C for 6 h with shaking at 200 rpm to allow dye adsorption to reach equilibrium. The absorbance of the supernatant solution after centrifugation was obtained on a Lambda 35 UV–Vis spectrophotometer at 410 and 599 nm, which represent the wavelengths of maximum absorbance for DO and DB, respectively. The amount of each dye adsorbed by the biomass sample was determined using the difference between the concentration of the initial added dye and the concentration of the dye in the supernatant. The maximum amount of DO or DB adsorbed to the substrate was calculated from the slope of a plot of *C*/*A* versus *C* according to the Langmuir adsorption equation shown below:

$$ \frac{C}{A} = \frac{1}{{K \times A_{m} }} + \frac{C}{{A_{m} }}, $$

where *C* (mg/mL) is the concentration of free dye in supernatant, *A* (mg/g biomass) is the amount of dye adsorbed by the substrate, *K* (mL/mg) is the Langmuir adsorption constant, and *A*_m_ (mg/g biomass) is the maximum amount of dye adsorbed.

### Statistical analysis and plotting

All statistical analyses were performed with Microsoft Excel 2010 and/or Origin Pro software and all figures were created using Origin Pro software. All samples representing transgenic or control lines (year 2 control, year 2 transgenic, year 3 control, and year 3 transgenic) were pair-compared (e.g., transgenic vs control and year 2 vs year 3) for such samples sets were made with parametric tests (e.g., Student’s *t* test for comparison of two means). The *t* test was applied with equal or unequal variances based on the results of an F test for each comparison. A one-tail *P* < 0.05 indicates that changes between compared groups were significant at 95% confidence level. The correlations between measured traits and sugar release were made across samples from all transgenic lines (10) and controls (5) in both years 2 and 3 by linear fitting. A Pearson coefficient was also calculated for guidance (although the Pearson correlation remains a consistent estimator of linearity, the test of its significance cannot be trusted without normality assumption).

## Additional file

**Additional file 1.** Supporting figures and tables of understanding the reduced cell wall recalcitrance of field-grown COMT Down-regulated switchgrass. **Figure S1.** Scheme of proposed lignin biosynthesis involving COMT. **Figure S2.** Relationship of cellulose DP to glucose release efficiency of unpretreated switchgrass. **Figure S3.** Relationship of hemicellulose molecular weights to xylose release efficiency of unpretreated switchgrass. **Figure S4.** Relationship of Cellulose crystallinity (CrI) to glucose release unpretreated switchgrass. **Table S1.** Original data used for Fig. 1: Chemical composition (per g cell wall residue) of field-grown switchgrass in years 2 and 3. The values reported are the average of 5 biological replicates from each control group, and 10 biological replicates from each transgenic group. Student *t* test was used as a statistical analysis for the difference between the transgenic and control groups. **Table S2.** Data used for Fig. 2a and b: Sugar release (mg per g cell wall residues) from hydrothermally pretreated (a) and unpretreated (b) switchgrass in years 2 and 3 after 72 h enzymatic hydrolysis (the value reported is the average of 5 biological replicates from each control group, and 10 biological replicates from each transgenic group). Student *t* test was used as a statistical analysis for the difference between the transgenic and control groups. **Table S3.** Original data used for Fig. 2c: the relationship of total sugar (glucose and xylose) release for pretreated switchgrass from enzymatic hydrolysis to lignin content (wt% of cell wall residues). **Table S4.** Original data used for Fig. 2d: the relationship of total sugar (glucose and xylose) release for unpretreated switchgrass from enzymatic hydrolysis to lignin content (wt% of cell wall residues). **Table S5.** Original data used for Figs. 3 and 4: distribution of DO adsorption and relationship of DO its relationship to sugar release (Fig. 3). Hemicellulose molecular weight distribution and Cellulose crystallinity index (CrI) (Fig. 4). Student’s *t* test was used as a statistical analysis for the difference between the transgenic and control groups. **Table S6.** Student’s *t* test results of traits difference in field-grown switchgrass between year 2 and 3.

## Abbreviations

A_O_: maximum adsorption of orange dye
*COMT*: caffeic acid *O*-methyltransferase
CP/MAS: cross-polarization magic angle spinning
DB: direct blue dye
CrI: crystallinity index
DO: direct orange dye
DP_n_: number-average degree of polymerization
DP_w_: weight-average degree of polymerization
M_n_: number-average molecular weight
M_w_: weight-average molecular weight
Py-MBMS: pyrolysis molecular beam mass spectrometry
