# Supplementary material for: Study of traits and recalcitrance reduction of field-grown COMT down-regulated switchgrass
Source: Biotechnol Biofuels. 2017 Jan 3;10:12. doi: 10.1186/s13068-016-0695-7 (PMC5209956; doi:10.1186/s13068-016-0695-7)
Supplement: Supplementary file 1 — Additional file 1. Supporting figures and tables of understanding the reduced cell wall recalcitrance of field-grown COMT Down-regulated switchgrass. Figure S1. Scheme of proposed lignin biosynthesis involving COMT. Figure S2. Relationship of cellulose DP to glucose release efficiency of unpretreated switchgrass. Figure S3. Relationship of hemicellulose molecular weights to xylose release efficiency of unpretreated switchgrass. Figure S4. Relationship of Cellulose crystallinity (CrI) to glucose release unpretreated switchgrass. Table S1. Original data used for Fig. 1: Chemical composition (per g cell wall residue) of field-grown switchgrass in years 2 and 3. The values reported are the average of 5 biological replicates from each control group, and 10 biological replicates from each transgenic group. Student t test was used as a statistical analysis for the difference between the transgenic and control groups. Table S2. Data used for Fig. 2a and b: Sugar release (mg per g cell wall residues) from hydrothermally pretreated (a) and unpretreated (b) switchgrass in years 2 and 3 after 72 h enzymatic hydrolysis (the value reported is the average of 5 biological replicates from each control group, and 10 biological replicates from each transgenic group). Student t test was used as a statistical analysis for the difference between the transgenic and control groups. Table S3. Original data used for Fig. 2c: the relationship of total sugar (glucose and xylose) release for pretreated switchgrass from enzymatic hydrolysis to lignin content (wt% of cell wall residues). Table S4. Original data used for Fig. 2d: the relationship of total sugar (glucose and xylose) release for unpretreated switchgrass from enzymatic hydrolysis to lignin content (wt% of cell wall residues). Table S5. Original data used for Figs. 3 and 4: distribution of DO adsorption and relationship of DO its relationship to sugar release (Fig. 3). Hemicellulose molecular weight distribution and Cellulose crystall [file 13068_2016_695_MOESM1_ESM.docx]

**Electronic Supplementary Information of Manuscript**

**Study of Traits and Recalcitrance Reduction of Field-grown *COMT* Down-regulated Switchgrass**

*Mi Li ^1,2,3^, Yunqiao Pu ^1,2,3^, Chang Geun Yoo ^1,2,3^, Erica Gjersing ^4^, Stephen R. Decker ^4^, Crissa Doeppke ^4^, Todd Shollengerger ^4^, Timothy J. Tschaplinski ^1,2,3^, Nancy L. Engle ^1,2,3^, Robert W. Sykes ^5^, Mark F. Davis ^5^, Holly L. Baxter ^1,6^, Mitra Mazarei ^1,6^, Chunxiang Fu ^7^, Richard A. Dixon ^1,8^,* *Zeng-Yu Wang ^1,7^, C. Neal Stewart, Jr. ^1,6^, Arthur J. Ragauskas ^*1,2,3,9^*

*^1^ BioEnergy Science Center (BESC), Oak Ridge National Laboratory (ORNL), Oak Ridge, TN, USA*

*^2^ BioSciences Division, ORNL, Oak Ridge, TN, USA*

*^3^ UT-ORNL Joint Institute for Biological Sciences, Oak Ridge, TN, USA*

*^4^ Biosciences Center, National Renewable Energy Laboratory (NREL), Golden, CO, USA*

*^5^ National Bioenergy Center, NREL, Golden, CO, USA*

*^6^ Department of Plant Sciences, University of Tennessee, Knoxville, TN, USA*

*^7^ Forage Improvement Division, The Samuel Roberts Noble Foundation, Ardmore, OK, USA*

*^8^ BioDiscovery Institute and Department of Biological Sciences, University of North Texas, Denton, TX, USA*

*^9^ Department of Chemical and Biomolecular Engineering & Department of Forestry, Wildlife, and Fisheries, University of Tennessee, Knoxville, TN, USA*

Figure S1. Scheme of proposed lignin biosynthesis involving COMT ^1-4^. PAL, phenylalanine ammonia-lyase; C4H, cinnamate 4-hydroxylase; C3H, p-coumarate 3-hydroxylase; CCoA-OMT, caffeoyl-CoA O-methyltransferase; HCT, 4-hydroxylcinnamoyl-CoA:quinate/shikimate 4-hydroxycinnamoyltransferease; COMT, caffeic acid O-methyltransferase; F5H, ferulate 5-hydroxylase; 4CL, 4-coumarate:CoA ligase; CCR, cinnamoyl-CoA reductase; CAD, cinnamyl alcohol dehydrogenase; H, p-hydroxyphenyl; C, catechyl; G, guaiacyl; 5-OH-G, 5-hydroxy-guaiacyl; S, syringyl.

Figure S2. Correlation of the number-average (DPn) and weight-average (DPw) degree of polymerization of cellulose with the glucose release efficiency of unpretreated switchgrass.

Figure S3. Correlation of the number-average (Mn) and weight-average (Mw) molecular weight of hemicellulose with the xylose release efficiency of unpretreated switchgrass.

Figure S4. Correlation of the crystallinity index (CrI) of cellulose with the glucose release efficiency of unpretreated switchgrass.

Table S1. Data used for Figure 1: Chemical composition (weight percentage of cell wall residue) of field-grown switchgrass in years 2 and 3. The values reported are the average of 5 biological replicates from each control group, and 10 biological replicates from each transgenic group. The second part of table below for detailed values for each group used for Figure 1B, 1C, 1D, and Student’s *t*-test between the transgenic and control groups.

|  | Year-2 control | | Year-2 transgenic | | Year-3 control | | Year-3 transgenic | |
| --- | --- | --- | --- | --- | --- | --- | --- | --- |
| (%) | **content** | **std-error** | **content** | **std-error** | **content** | **std-error** | **content** | **std-error** |
| Arabinan | 2.41 | 0.10 | 2.56 | 0.07 | 2.28 | 0.07 | 2.39 | 0.08 |
| Galactan | 1.35 | 0.03 | 1.46 | 0.02 | 1.40 | 0.06 | 1.52 | 0.04 |
| Glucan | 36.41 | 0.53 | 37.37 | 0.86 | 38.41 | 0.34 | 38.84 | 0.31 |
| Xylan | 22.67 | 0.28 | 24.58 | 0.56 | 21.99 | 0.35 | 23.58 | 0.19 |
| Mannan | ND | ND | ND | ND | ND | ND | ND | ND |
| Lignin | 25.31 | 0.25 | 22.38 | 0.12 | 27.48 | 0.18 | 23.71 | 0.23 |

ND: not detectable; N/A: not applicable; std-error: standard error.

|  | Year 2 | | | | |  | Year 3 | | | | |
| --- | --- | --- | --- | --- | --- | --- | --- | --- | --- | --- | --- |
|  | **Arabinan** | **Galactan** | **Glucan** | **Xylan** | **Lignin** |  | **Arabinan** | **Galactan** | **Glucan** | **Xylan** | **Lignin** |
|  | **%** | **%** | **%** | **%** | **%** | **1** | **%** | **%** | **%** | **%** | **%** |
| Control 1 | 2.46 | 1.40 | 37.78 | 22.97 | 24.3 |  | 2.26 | 1.38 | 38.49 | 21.35 | 25.77 |
| Control 2 | 2.33 | 1.30 | 36.74 | 22.39 | 23.65 |  | 2.41 | 1.50 | 39.04 | 21.71 | 24.92 |
| Control 3 | 2.68 | 1.45 | 34.94 | 21.86 | 22.92 |  | 2.20 | 1.31 | 39.05 | 21.50 | 25.52 |
| Control 4 | 2.43 | 1.32 | 36.67 | 23.30 | 23.26 |  | 2.41 | 1.53 | 37.41 | 22.45 | 25.77 |
| Control 5 | 2.14 | 1.29 | 35.92 | 22.85 | 23.57 |  | 2.09 | 1.26 | 38.08 | 22.96 | 25.8 |
| Transgenic 1 | 2.51 | 1.44 | 38.42 | 24.33 | 20.66 |  | 2.06 | 1.40 | 39.77 | 23.55 | 22.6 |
| Transgenic 2 | 2.51 | 1.41 | 36.49 | 23.94 | 20.29 |  | 2.22 | 1.44 | 38.49 | 22.87 | 22.1 |
| Transgenic 3 | 2.45 | 1.39 | 37.91 | 24.71 | 20.57 |  | 2.34 | 1.40 | 39.14 | 24.05 | 21.43 |
| Transgenic 4 | 2.67 | 1.50 | 37.76 | 25.18 | 21.11 |  | 2.29 | 1.57 | 39.19 | 23.30 | 21.04 |
| Transgenic 5 | 2.61 | 1.46 | 36.23 | 23.82 | 21.05 |  | 2.41 | 1.50 | 38.40 | 23.43 | 21.61 |
| Transgenic 6 | 2.57 | 1.53 | 38.33 | 24.61 | 21.29 |  | 2.21 | 1.50 | 40.67 | 23.56 | 23.3 |
| Transgenic 7 | 2.38 | 1.43 | 35.34 | 23.13 | 20.56 |  | 2.59 | 1.68 | 38.14 | 22.95 | 21.37 |
| Transgenic 8 | 2.45 | 1.39 | 33.95 | 23.23 | 20.43 |  | 2.81 | 1.69 | 37.73 | 24.76 | 22.3 |
| Transgenic 9 | 3.08 | 1.59 | 43.01 | 28.62 | 21.05 |  | 2.63 | 1.57 | 38.24 | 23.74 | 22.21 |
| Transgenic 10 | 2.41 | 1.45 | 36.24 | 24.19 | 21.11 |  | 2.34 | 1.43 | 38.61 | 23.63 | 22.51 |
| P value | 0.09 | 0.01 | 0.21 | <0.01 | <0.0001 |  | 0.16 | 0.03 | 0.18 | <0.01 | <0.0001 |

Table S2. Data used for Figure 2A and 2B: Sugar release (mg per g cell wall residues) from hydrothermally pretreated (A) and unpretreated (B) switchgrass in year 2 and year 3 after 72 h enzymatic hydrolysis (the value reported is the average of 5 biological replicates from each control group, and 10 biological replicates from each transgenic group). Student’s *t*-test was used as a statistical analysis for the difference between the transgenic and control groups.

|  | Glc | std-error | *P*-value | Xyl | std-error | *P*-value | Total | std-error | *P*-value |
| --- | --- | --- | --- | --- | --- | --- | --- | --- | --- |
| Hydrothermally pretreated | | | | | | | | | |
| Year-2 control | 126.49 | 4.37 |  | 163.01 | 2.17 |  | 289.50 | 4.83 |  |
| Year-2 transgenic | 181.54 | 3.98 | <0.0001 | 200.46 | 2.64 | <0.0001 | 382.00 | 6.34 | <0.0001 |
| Year-3 control | 131.68 | 3.43 |  | 169.40 | 2.24 |  | 301.08 | 3.89 |  |
| Year-3 transgenic | 182.26 | 10.19 | <0.001 | 193.10 | 5.93 | <0.001 | 375.36 | 15.73 | <0.001 |
| Unpretreated | | | | | | | | | |
| Year-2 control | 19.52 | 1.44 |  | 8.90 | 0.30 |  | 28.42 | 1.51 |  |
| Year-2 transgenic | 25.05 | 0.90 | <0.0001 | 14.14 | 0.50 | <0.0001 | 39.18 | 1.23 | <0.0001 |
| Year-3 control | 17.49 | 0.93 |  | 8.31 | 0.61 |  | 25.80 | 1.32 |  |
| Year-3 transgenic | 31.49 | 2.13 | <0.0001 | 16.04 | 0.54 | <0.0001 | 47.53 | 2.63 | <0.0001 |

Glc: glucose; Xyl: xylose; Total: sum of glucose and xylose; std-error: standard error.

Table S3. Data used for Figure 2C: the relationship of total sugar (glucose and xylose) release for pretreated (C) switchgrass from enzymatic hydrolysis to lignin content (wt% of cell wall residues).

|  | Year-2 | | | | | | |  | Year-3 | | | | | | |
| --- | --- | --- | --- | --- | --- | --- | --- | --- | --- | --- | --- | --- | --- | --- | --- |
|  | **Lignin** | **Total** |  | **Glc** |  | **Xyl** |  |  | **Lignin** | **Total** |  | **Glc** |  | **Xyl** |  |
|  | **%** | **mg/g** | **stdev** | **mg/g** | **stdev** | **mg/g** | **stdev** |  | **%** | **mg/g** | **stdev** | **mg/g** | **stdev** | **mg/g** | **stdev** |
| Control 1 | 24.30 | 290.49 | 7.60 | 126.24 | 2.42 | 164.24 | 6.30 |  | 25.77 | 302.72 | 10.70 | 138.96 | 11.42 | 163.76 | 5.16 |
| Control 2 | 23.65 | 276.93 | 8.88 | 122.12 | 6.63 | 154.80 | 2.72 |  | 24.92 | 313.86 | 15.17 | 137.01 | 8.59 | 176.85 | 7.00 |
| Control 3 | 22.92 | 305.46 | 14.19 | 142.58 | 10.25 | 162.88 | 4.14 |  | 25.52 | 302.12 | 30.69 | 135.53 | 19.52 | 166.59 | 11.23 |
| Control 4 | 23.26 | 291.97 | 34.04 | 125.10 | 20.75 | 166.88 | 13.58 |  | 25.77 | 290.47 | 7.24 | 122.08 | 8.31 | 168.39 | 1.70 |
| Control 5 | 23.57 | 282.65 | 10.45 | 116.41 | 5.04 | 166.24 | 5.47 |  | 25.80 | 296.23 | 16.67 | 124.81 | 11.99 | 171.42 | 6.95 |
| Transgenic 1 | 20.66 | 375.25 | 2.86 | 180.76 | 3.16 | 194.48 | 2.31 |  | 22.60 | 310.76 | 64.32 | 142.00 | 40.80 | 168.76 | 25.16 |
| Transgenic 2 | 20.29 | 391.56 | 5.61 | 187.72 | 3.61 | 203.84 | 2.33 |  | 22.10 | 405.03 | 27.43 | 206.06 | 23.30 | 198.97 | 4.18 |
| Transgenic 3 | 20.57 | 383.73 | 19.56 | 183.34 | 7.51 | 200.39 | 12.09 |  | 21.43 | 402.74 | 18.41 | 198.39 | 14.19 | 204.35 | 5.08 |
| Transgenic 4 | 21.11 | 375.54 | 13.18 | 172.19 | 10.27 | 203.35 | 4.15 |  | 21.04 | 398.98 | 21.14 | 196.42 | 19.43 | 202.56 | 2.78 |
| Transgenic 5 | 21.05 | 403.78 | 51.89 | 196.85 | 28.16 | 206.93 | 24.03 |  | 21.61 | 359.69 | 34.70 | 170.52 | 20.51 | 189.17 | 14.28 |
| Transgenic 6 | 21.29 | 331.76 | 10.08 | 152.73 | 6.60 | 179.03 | 3.50 |  | 23.30 | 272.01 | 37.96 | 115.57 | 21.38 | 156.44 | 16.85 |
| Transgenic 7 | 20.56 | 396.04 | 13.28 | 192.74 | 6.98 | 203.30 | 6.42 |  | 21.37 | 399.17 | 25.32 | 210.06 | 25.12 | 189.12 | 1.37 |
| Transgenic 8 | 20.43 | 389.92 | 19.63 | 182.46 | 8.04 | 207.46 | 12.11 |  | 22.30 | 433.10 | 30.59 | 213.25 | 19.49 | 219.85 | 11.34 |
| Transgenic 9 | 21.05 | 394.53 | 25.81 | 190.28 | 23.51 | 204.25 | 3.48 |  | 22.21 | 406.73 | 13.59 | 198.55 | 8.57 | 208.18 | 12.15 |
| Transgenic 10 | 21.11 | 377.88 | 17.98 | 176.34 | 5.18 | 201.54 | 13.72 |  | 22.51 | 365.39 | 21.24 | 171.78 | 12.42 | 193.61 | 9.25 |

Glc: glucose; Xyl: xylose; Total: sum of glucose and xylose; stdev: standard deviation of duplicate.

Table S4. Data used for Figure 2D: the relationship of total sugar (glucose and xylose) release for unpretreated (D) switchgrass from enzymatic hydrolysis to lignin content (wt% of cell wall residues).

|  | Year-2 | | | | | | |  | Year-3 | | | | | | |
| --- | --- | --- | --- | --- | --- | --- | --- | --- | --- | --- | --- | --- | --- | --- | --- |
|  | **Lignin** | **Total** |  | **Glc** |  | **Xyl** |  |  | **Lignin** | **Total** |  | **Glc** |  | **Xyl** |  |
|  | **%** | **mg/g** | **stdev** | **mg/g** | **stdev** | **mg/g** | **stdev** |  | **%** | **mg/g** | **stdev** | **mg/g** | **stdev** | **mg/g** | **stdev** |
| Control 1 | 24.30 | 26.37 | 6.49 | 18.17 | 4.34 | 8.19 | 2.27 |  | 25.77 | 28.61 | 1.48 | 18.17 | 1.18 | 10.45 | 1.21 |
| Control 2 | 23.65 | 29.94 | 2.48 | 20.17 | 1.80 | 9.77 | 1.09 |  | 24.92 | 26.59 | 3.12 | 18.67 | 1.11 | 7.91 | 2.13 |
| Control 3 | 22.92 | 33.45 | 3.75 | 24.78 | 3.30 | 8.67 | 0.57 |  | 25.52 | 27.33 | 3.65 | 19.48 | 1.99 | 7.85 | 2.17 |
| Control 4 | 23.26 | 27.52 | 4.04 | 18.12 | 2.81 | 9.40 | 1.34 |  | 25.77 | 20.90 | 1.58 | 14.16 | 0.76 | 6.74 | 0.87 |
| Control 5 | 23.57 | 24.84 | 1.43 | 16.38 | 1.70 | 8.46 | 1.57 |  | 25.80 | 25.56 | 3.39 | 16.95 | 1.88 | 8.61 | 1.65 |
| Transgenic 1 | 20.66 | 41.73 | 2.08 | 26.11 | 2.28 | 15.63 | 0.40 |  | 22.60 | 37.79 | 1.94 | 23.33 | 0.98 | 14.46 | 1.56 |
| Transgenic 2 | 20.29 | 43.18 | 4.89 | 27.26 | 2.75 | 15.92 | 2.15 |  | 22.10 | 51.52 | 7.55 | 33.89 | 5.24 | 17.62 | 2.33 |
| Transgenic 3 | 20.57 | 36.91 | 2.72 | 22.62 | 2.28 | 14.28 | 0.48 |  | 21.43 | 54.69 | 0.85 | 36.96 | 2.76 | 17.73 | 1.92 |
| Transgenic 4 | 21.11 | 45.36 | 10.45 | 31.30 | 8.23 | 14.06 | 2.30 |  | 21.04 | 56.79 | 12.12 | 39.68 | 10.91 | 17.11 | 1.44 |
| Transgenic 5 | 21.05 | 38.22 | 2.48 | 23.95 | 2.63 | 14.27 | 0.54 |  | 21.61 | 52.21 | 4.81 | 35.12 | 4.47 | 17.09 | 0.35 |
| Transgenic 6 | 21.29 | 31.94 | 11.49 | 21.48 | 7.74 | 10.46 | 3.84 |  | 23.30 | 36.25 | 6.07 | 22.69 | 2.78 | 13.56 | 3.30 |
| Transgenic 7 | 20.56 | 41.80 | 1.18 | 26.58 | 1.63 | 15.22 | 0.55 |  | 21.37 | 57.16 | 3.89 | 38.97 | 2.40 | 18.19 | 1.73 |
| Transgenic 8 | 20.43 | 36.66 | 4.91 | 23.16 | 4.13 | 13.50 | 1.30 |  | 22.30 | 36.06 | 0.91 | 21.92 | 0.42 | 14.15 | 0.76 |
| Transgenic 9 | 21.05 | 38.92 | 3.89 | 23.92 | 1.50 | 15.00 | 2.44 |  | 22.21 | 45.70 | 0.73 | 31.07 | 0.72 | 14.63 | 0.05 |
| Transgenic 10 | 21.11 | 37.12 | 2.02 | 24.09 | 2.84 | 13.03 | 2.06 |  | 22.51 | 47.17 | 2.56 | 31.30 | 0.97 | 15.87 | 1.61 |

Glc: glucose; Xyl: xylose; Total: sum of glucose and xylose; stdev: standard deviation of duplicate.

Table S5. Data used for Figure 3, and 4: Distibution of direct orange (DO) adsorption (Figure 3A); Total sugar release (glucose and xylose) for unpretreated switchgrass from enzymatic hydrolysis and their relationship to direct orange (DO) adsorption measured by Simons’ stain (Figure 3B); Distribution of hemicellulose Mw (Figure 5A); and the average cellulose crystallinity index (CrI). Student’s *t*-test was used as a statistical analysis for the difference between the transgenic and control groups.

|  | Year-2 | | | | | | | |  | Year-3 | | | | | | | |
| --- | --- | --- | --- | --- | --- | --- | --- | --- | --- | --- | --- | --- | --- | --- | --- | --- | --- |
|  | **Total** | **DO** | **lignin** | **DPn** | **DPw** | **Mn** | **Mw** | **CrI** |  | **Total** | **DO** | **lignin** | **DPn** | **DPw** | **Mn** | **Mw** | **CrI** |
|  | **mg/g** | **mg/g** | **%** |  |  | **g/mol** | **g/mol** |  |  | **mg/g** | **mg/g** | **%** |  |  | **g/mol** | **g/mol** |  |
| Control 1 | 26.37 | 5.97 | 24.30 | 426 | 3902 | 16553 | 27379 | 0.39 |  | 28.61 | 5.96 | 25.77 | 386 | 3665 | 22737 | 35379 | 0.36 |
| Control 2 | 29.94 | 5.56 | 23.65 | 392 | 3461 | 20102 | 30344 | 0.41 |  | 26.59 | 5.98 | 24.92 | 502 | 3962 | 23926 | 37252 | 0.39 |
| Control 3 | 33.45 | 5.32 | 22.92 | 439 | 3706 | 21515 | 31474 | 0.36 |  | 27.33 | 5.92 | 25.52 | 440 | 3988 | 24034 | 37316 | 0.38 |
| Control 4 | 27.52 | 6.00 | 23.26 | 438 | 3677 | 26322 | 35418 | 0.37 |  | 20.90 | 5.06 | 25.77 | 408 | 3802 | 22788 | 35365 | 0.38 |
| Control 5 | 24.84 | 3.92 | 23.57 | 460 | 3899 | 23490 | 33304 | 0.36 |  | 25.56 | 3.82 | 25.80 | 372 | 3824 | 24888 | 34764 | 0.40 |
| Transgenic 1 | 41.73 | 6.52 | 20.66 | 506 | 3975 | 22655 | 32173 | 0.35 |  | 37.79 | 6.31 | 22.60 | 400 | 3781 | 21799 | 33899 | 0.36 |
| Transgenic 2 | 43.18 | 7.34 | 20.29 | 481 | 3821 | 23711 | 31701 | 0.36 |  | 51.52 | 7.48 | 22.10 | 412 | 3848 | 20780 | 32966 | 0.37 |
| Transgenic 3 | 36.91 | 7.05 | 20.57 | 428 | 4304 | 19198 | 28684 | 0.36 |  | 54.69 | 6.27 | 21.43 | 388 | 3814 | 19508 | 29368 | 0.38 |
| Transgenic 4 | 45.36 | 7.55 | 21.11 | 491 | 3983 | 18207 | 27220 | 0.34 |  | 56.79 | 6.03 | 21.04 | 368 | 3797 | 20004 | 31265 | 0.37 |
| Transgenic 5 | 38.22 | 6.10 | 21.05 | 345 | 3157 | 17522 | 26860 | 0.37 |  | 52.21 | 8.33 | 21.61 | 340 | 3884 | 20423 | 30899 | 0.39 |
| Transgenic 6 | 31.94 | 5.47 | 21.29 | 469 | 3785 | 16925 | 27344 | 0.33 |  | 36.25 | 6.09 | 23.30 | 499 | 4147 | 20836 | 31528 | 0.36 |
| Transgenic 7 | 41.80 | 6.12 | 20.56 | 432 | 3839 | 19390 | 29033 | 0.37 |  | 57.16 | 6.77 | 21.37 | 383 | 3745 | 20079 | 26769 | 0.39 |
| Transgenic 8 | 36.66 | 8.23 | 20.43 | 432 | 3909 | 18992 | 28886 | 0.38 |  | 36.06 | 5.30 | 22.30 | 416 | 3768 | 20296 | 31000 | 0.39 |
| Transgenic 9 | 38.92 | 5.51 | 21.05 | 417 | 3693 | 20777 | 30147 | 0.34 |  | 45.70 | 5.89 | 22.21 | 439 | 4276 | 23367 | 36845 | 0.38 |
| Transgenic 10 | 37.12 | 6.05 | 21.11 | 380 | 4081 | 20046 | 30549 | 0.37 |  | 47.17 | 5.91 | 22.51 | 370 | 3695 | 24353 | 36228 | 0.39 |
| P-value | 0.00 | 0.01 | 0.00 | 0.39 | 0.20 | 0.12 | 0.04 | 0.05 |  | 0.00 | 0.02 | 0.00 | 0.23 | 0.39 | 0.00 | 0.00 | 0.23 |

Table S6. Student’s *t*-test (one-tail *P* value) of traits comparison between unpretreated year 2 and year 3 switchgrass.

|  | Year 2 vs year 3  control | Year 2 vs year 3  transgenic |
| --- | --- | --- |
| Arabinan | 0.13 | 0.04 |
| Galactan | 0.25 | 0.07 |
| Glucan | 0.004 | 0.05 |
| Xylan | 0.06 | 0.05 |
| Mannan | N/A | N/A |
| Lignin | <0.0001 | <0.0001 |
| A_O_ | 0.49 | 0.35 |
| Hemicellulose Mw | 0.01 | 0.01 |
| Hemicellulose Mn | 0.14 | 0.06 |
| Cellulose DPw | 0.13 | 0.43 |
| Cellulose DPn | 0.36 | 0.05 |
| Cellulose CrI | 0.37 | <0.01 |

1 Guo, D., Chen, F., Inoue, K., Blount, J. W. & Dixon, R. A. Downregulation of caffeic acid 3-O-methyltransferase and caffeoyl CoA 3-O-methyltransferase in transgenic alfalfa: impacts on lignin structure and implications for the biosynthesis of G and S lignin. *The Plant Cell* **13**, 73-88 (2001).

2 Boerjan, W., Ralph, J. & Baucher, M. Lignin biosynthesis. *Annual Review of Plant Biology* **54**, 519-546 (2003).

3 Chen, F., Tobimatsu, Y., Havkin-Frenkel, D., Dixon, R. A. & Ralph, J. A polymer of caffeyl alcohol in plant seeds. *Proc. Natl. Acad. Sci.* **109**, 1772-1777 (2012).

4 Vanholme, R., Demedts, B., Morreel, K., Ralph, J. & Boerjan, W. Lignin biosynthesis and structure. *Plant Physiology* **153**, 895-905 (2010).
